# Intestinal failure in kaposiform hemangioendothelioma successfully resolved with medical therapy and nearly fat-free oral feeding: A novel case report

**DOI:** 10.1016/j.intf.2025.100347

**Published:** 2026-01-14

**Authors:** Tierra L. Mosher, Eduardo J. Contijoch, Jordan Bui, Joyce Teng, Michael R. Jeng, Shweta S. Namjoshi

**Affiliations:** a Stanford Medicine Children’s Health, Palo Alto, CA 94304, United States; b Georgetown University School of Medicine, Washington, DC 20007, United States; c Stanford University School of Medicine, Department of Dermatology, Palo Alto, CA 94304, United States; d Stanford University School of Medicine, Department of Pediatrics, Palo Alto, CA 94304, United States; e Stanford University School of Medicine, Division of Gastroenterology Hepatology & Nutrition, Palo Alto, CA 94304, United States

**Keywords:** fat-free diet, abdominal compartment syndrome, Kaposiform hemangioendothelioma, Lymphatic malformation, Protein losing enteropathy

## Abstract

**Background:**

Kaposiform hemangioendothelioma and kaposiform lymphangiomatosis (KHE/KLA) are rare, non-malignant lymphovascular anomalies often complicated by Kasabach-Merritt phenomenon (KMP). Morbidity and mortality are high. There are no prior reports of the dietary management in these disorders.

**Case report:**

A 2-month-old female presented with hemorrhagic ascites, abdominal compartment syndrome, and severe KMP secondary to extensive retroperitoneal and intestinal KHE/KLA. Following emergent surgical management for abdominal decompression and diverting ileostomy, medical and nutritional therapy with steroids, vincristine, sirolimus, and parenteral nutrition were employed for management. After maintaining a near fat-free enteral diet supplemented with intravenous lipid therapy in the outpatient setting, she underwent successful ileostomy takedown 18 months after initial diagnosis without the need for intestinal resection and maintenance of adequate growth.

**Conclusion:**

This case underscores the value of clinical and pathology-guided diagnosis, coordinated medical therapy, and dietary fat restriction for achieving intestinal autonomy and preserving bowel length in intra-abdominal KHE/KLA.

## Introduction

Kaposiform hemangioendothelioma (KHE)/ Kaposiform Lymphangiomatosis (KLA) are rare, locally aggressive though non-malignant lymphovascular tumors of childhood that develop in the deep soft tissue of the extremities, head and neck, retroperitoneum, mediastinum, bone, or other organs [1], [2]. The tumors most commonly present in children less than one year of age, with incidence decreasing with older age [2].

KHE/KLA can be associated with severe morbidity and mortality, and up to 70 % of patients experience Kasabach-Merritt phenomenon (KMP) with thrombocytopenia and coagulopathy [2]. KMP can be fatal in up to 30 % of patients with KHE/KLA and can lead to intracranial bleeding, disseminated intravascular coagulation, and organ compartment syndrome [2]. Moreover, despite early intervention being critical to preventing tumor progression, there have been limited described treatment options for KHE/KLA with KMP outside of surgical resection, especially in the case of intraabdominal involvement [1], [2], [3].

We highlight the case of an infant with KHE/KLA and recurring KMP who was critically ill with a fatal trajectory. Using targeted therapy and fat restriction, the infant achieved near enteral autonomy relying on intravenous multicomposite fat emulsion (SMOFLipid®) and otherwise exclusive oral feeding.

## Case report

**Fig. 1:**
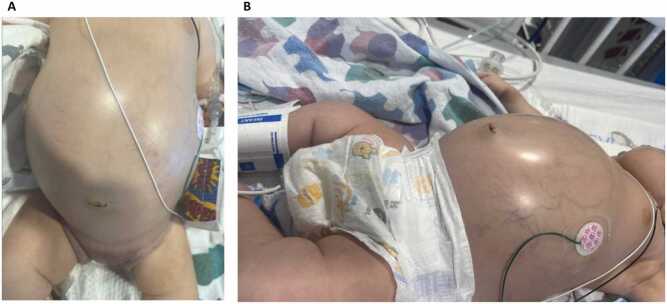
Initial physical exam findings of infantile kaposiform hemangioendothelioma and kaposiform lymphangiomatosis. A and B both highlight a large, distended abdomen that was tender to palpation with quiet bowel sounds. A also highlights a vulvar rash to include swollen ecchymosis of the labia, with no other areas of bruising or skin lesions.

A previously healthy 38-week gestation female presented to the emergency department with watery diarrhea and a petechial perineal rash at two months of age. A presumed diagnosis of viral gastroenteritis complicated by diaper rash was made and a soy-based formula was trialed. There was transient improvement in stools on this formula, but she returned to the emergency room with bloody stools, tense violaceous abdominal distension, and labial ecchymosis (Fig. 1). Laboratory studies showed hemoglobin 4.9 g/dL, platelets 7000 K/uL, reticulocytosis of 21 %, sodium 126 mmol/L, and albumin 3.4 g/dL. Infectious and hemolysis labs were unremarkable. Abdominal ultrasound demonstrated free fluid without organomegaly. A contrast computed tomography (CT) study confirmed massive ascites. She was admitted to the pediatric intensive care unit (PICU) (Hospital Day (HD) 0).

Stabilization required packed red blood cells and platelet transfusions with peritoneal drainage. Despite these measures, she developed abdominal compartment syndrome culminating in cardiac arrest on HD 14. Exploratory laparotomy revealed large-volume serous ascites and hepatomegaly with extramedullary hematopoiesis. Empiric eculizumab for presumed atypical hemolytic uremic syndrome (HUS) was administered without improvement.

**Fig. 2:**
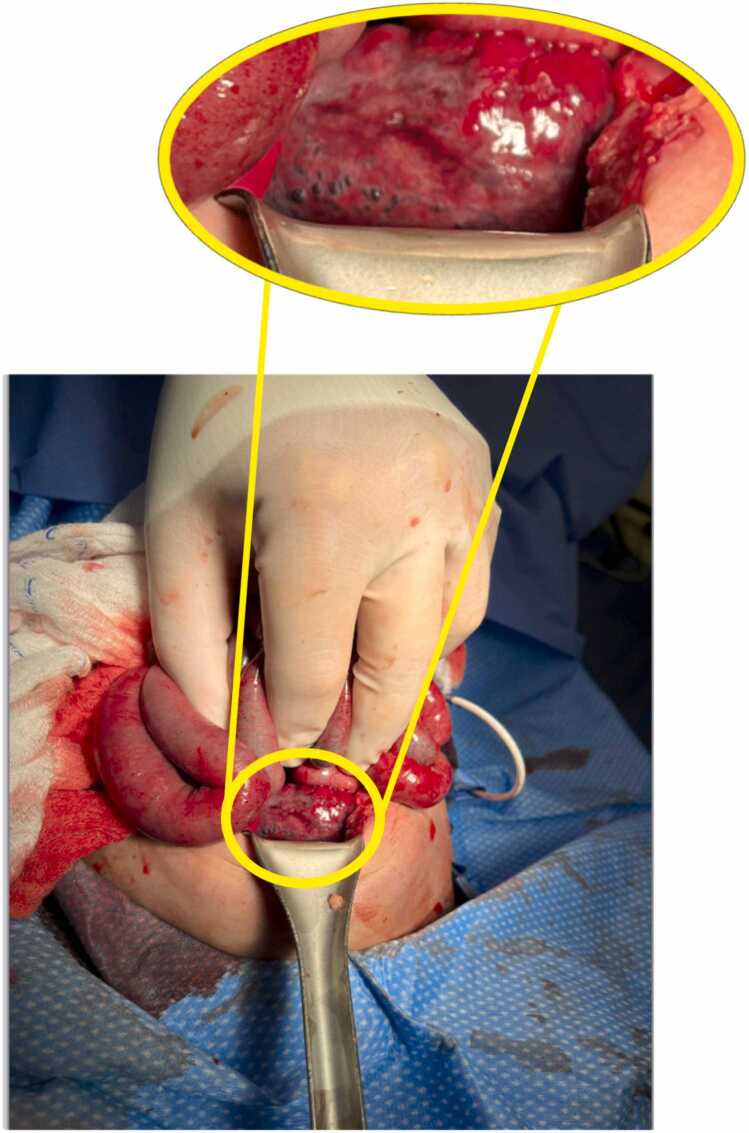
Gross intraoperative findings of infantile kaposiform hemangioendothelioma and kaposiform lymphangiomatosis. This highlights pertinent findings during the patient’s exploratory laparotomy: the sigmoid colon is shown with enhancement of the yellow circled area to highlight ‘blue dots’ more clearly which represent engorged purple capillaries on the serosa in a gray-purple, reticular pattern. The intestine is swollen but not necrotic.

A repeat exploratory laparotomy demonstrated rubber-hose-like thickened colon with gray-purple serosal lesions and thrombosed mesenteric vessels (Fig. 2). Overall, the lesion demonstrated diffuse retroperitoneal involvement with associated intestinal disease, characterized by colonic serosal lesions and thrombosed mesenteric vessels. A proximal diversion with end ileostomy was created. Biopsies from labial lesion showed a proliferation of endothelial cells with lymphatic phenotype as well as spindle-cell vascular tufts positive for D2–40 and CD31, consistent with KHE/KLA. Blood samples and tissue from the labial lesion were submitted for genetic testing, and no clinically relevant sequence variants were identified. Additional next generation sequencing-based mutational profiling using the Stanford Actionable Mutation Panel (STAMP) for solid tumors was performed as well. STAMP found no pathogenic or likely pathogenic variants identified, although there was a low percentage of lesional tissue content which may adversely affect the assay's sensitivity. Of note, angiopoietin-2 was also submitted at the same time with a result of 13,900 pg/mL (reference range 1434 – 4141 pg/mL). Elevated angiopoietin-2 has been observed in both KHE and KLA, especially in those patients with coagulopathy [4]. It has also been used as a biomarker to follow response to therapy [4]. High-dose methylprednisolone (30 mg/kg/day) and sirolimus (target trough 8–12 ng/mL) were initiated.

Serial abdominal washouts allowed fascial closure with biologic mesh. In the setting of high ileostomy output (∼ 67 mL/kg/day), the patient was placed on parenteral nutrition (PN) to deliver 65 % of calories. The remaining calories were provided through nasogastric tube with oral feeds for comfort. She transitioned from the PICU to the acute care floor where enteral feeds with hydrolyzed formula were advanced to 35 % of nasogastric tube intake, and the patient was discharged home on this combination of parenteral nutrition and high medium chain triglyceride formula feeds after five months (HD 150).

While outpatient, a three-month vincristine course with sirolimus enabled weaning of systemic steroids. Dynamic contrast magnetic resonance (MR) lymphangiography revealed tortuous thoracic duct and preferential retroperitoneal lymphatic filling, further supporting mixed KHE/KLA physiology. Serum angiopoietin-2 was elevated at 4989 pg/mL.

A brief attempt to reintroduce a standard-to high fat diet triggered recurrent thrombocytopenia and KMP mass enlargement and hospital admission, so a strict low-to-no fat oral feeding plan was reintroduced and re-enforced, with essential fatty acids and extra caloric density supplied through multicomposite fat emulsion at 2 g/kg/day via a silastic, single lumen, central venous catheter. Over the 18 months since the patient’s initial presentation, she has advanced starch, vegetable, and no fat protein-based solids, discontinued parenteral nutrition remaining only on multicomposite fat emulsion, and has maintained a weight-for-length z-score of −0.1 primarily through tube and oral feeds.

**Table 1:** Detailed timeline of clinical course of an infant with infantile kaposiform hemangioendothelioma and kaposiform lymphangiomatosis.

**Hospital Day (HD)**	**Event/Intervention**	**Key Diagnostics/Findings**	**Outcome/Notes**
**Pre-Admission**	**ED visit #1**	—	Symptoms initially attributed to viral syndrome
**HD0**	**ED visit #2**	**Hgb 4.9, Hct 15.8, Platelets 7, Retic 21 %**	Returned with rash, diarrhea, and worsening distension
		**Na 126, Cl 97, Alb 3.4**	
		No schistocytes on smear	
		Infection screen negative	
**HD0**	**Admission to PICU; RBC & platelet transfusions**	**CT Abd/Pelvis**: Diffuse ascites, periportal edema	Stabilized hemodynamically
**HD3**	**Placement of peritoneal drain (IR)**	∼200 mL serosanguinous fluid drained	—
**HD8**	**Flexible sigmoidoscopy & bone marrow biopsy**	Sigmoid congestion, 1 CMV inclusion	Ganciclovir started (no clinical improvement)
		Bone marrow normal	
**HD14**	**Cardiac arrest (hyperkalemia); 2nd IR drain placement**	Another ∼200 mL drained; patient stabilized	—
**HD15**	**Exploratory laparotomy & liver biopsy**	Large-volume ascites, pancolitis, normal small bowel	—
**HD18**	**Initiated eculizumab**	—	Empiric treatment for possible aHUS (no improvement)
**HD22**	**Repeat exploratory laparotomy**	Minimal fluid, “rubber hose” thickened colon	Abdomen left open with silo
**HD23**	**End ileostomy creation**	—	High-dose steroids & sirolimus started for KHE
**HD25**	**Skin biopsy (labia)**	Pathology consistent with KHE	—
**HD30, 32, 36**	**Sequential OR washouts & partial closures**	—	Final closure with biologic mesh on HD36
**HD49**	**Full enteral feeds attempt**	—	High ileostomy output (∼66 mL/kg/day) → partial TPN
**HD60–90**	**Transfer to GI service → Discharge**	—	65 % TPN/SMOF + 35 % oral/hydrolyzed formula
**3 months post-hospital discharge**	**IR lymphatic mapping**	Retroperitoneal & bowel involvement, thoracic duct anomalies, no discrete mass	—
**4 months post-hospital discharge**	**Vincristine (10-dose course)**	—	Bridged steroid wean; patient remains on sirolimus
**17 months post-hospital discharge**	**Ileostomy takedown and primary reanastomosis**	1.5:1 size mismatch noted	—
**Present day**	**Patient remains on sirolimus monotherapy with oral feeds + IV SMOFlipids**		

**Abbreviations**: **ED emergency room** Hgb, hemoglobin; Hct, hematocrit; Retic, reticulocyte count; Alb, albumin; IR, interventional radiology; Abd/Pelvis, abdomen/pelvis; aHUS, atypical hemolytic uremic syndrome; KHE, Kaposiform Hemangioendothelioma; TPN, total parenteral nutrition; SMOF, soybean oil, medium-chain triglycerides, olive oil, and fish oil.

Seventeen months after initial presentation to care, she underwent ileostomy takedown with primary ileal anastomosis. Pathology findings taken during the takedown were listed as consistent with ileostomy without signs of KHE/KLA present. She resumed her zero-fat diet within 48 h of surgery and has had no postoperative complications. There have been no active bleeding, KMP, nor tumor progression episodes to date, and she remains clinically well on sirolimus 1 mg/m^2^ twice daily as well as isolated multicomposite fat emulsion lipids. In addition, her angiopoietin-2 levels normalized and remain normal at this time.

## Discussion

Diffuse intra-abdominal KHE/KLA presents a therapeutic dilemma in which surgical resection may be curative yet risks catastrophic loss of bowel length and its associated long term complications including exclusive parenteral nutrition requirement, infections, anastomotic ulceration and/or leak, etc [5], [6]. Alternatively, conservative management must control KMP and excess lymphatic leak [7]. Sirolimus, an mTOR inhibitor, normalizes abnormal lymphangiogenesis and improves coagulopathy; corticosteroids reduce inflammation; and vincristine assists in inhibition of mitosis and induces apoptosis in tumor-derived cells, thereby targeting rapidly proliferating endothelial cells and halting that process [8], [9], [10], [11], [12], [13], [14]. In addition to highlighting this medical regimen, our case also highlights the importance of strict fat restriction complemented by pharmacotherapy to ensure success, which was an essential component in the success of this patient’s management, and not highlighted in the literature thus far. This enteral fat restriction requirement likely is required to reduce intestinal lymph flow, therefore managing chylous leak [7]. At the time of presentation, our team was advised not to feed this child to avoid fatal KMP, but we have demonstrated long term success with a no fat diet and plan to re-introduce low dose fat in the years to come if the lesion remains in remission with time.

Fewer than 10 cases of abdominal KHE/KLA have been reported, with most cases requiring bowel resection or resulting in mortality. Here, we report the first case successfully managed with preservation of intestinal length and progression to near-enteral autonomy through a combination of medical therapy and dietary fat restriction. A limitation of our report is lack of alpha-1 antitrypsin levels while on a high MCT formula, as our patient had an ileostomy while she was on partial fat containing formula feeding. Given recurring KMP on oral fat reintroduction, and subsequent fat restriction, we have not checked a stool alpha-1 antitrypsin status post ileostomy take-down. Her albumin levels have remained normal post take down.

We hope this early diagnosis of intra-abdominal KHE/KLA combined with optimal medical and dietary management can guide future practitioners in managing patients with this condition, allowing for preserved bowel and ultimately enteral autonomy.

## CRediT authorship contribution statement

**Joyce Teng:** Writing – review & editing. **Jordan Bui:** Writing – review & editing, Resources. **Eduardo J Contijoch:** Writing – review & editing, Investigation, Formal analysis. **Tierra L. Mosher:** Writing – review & editing, Writing – original draft, Resources, Project administration, Methodology, Investigation, Data curation. **Shweta S. Namjoshi:** Writing – review & editing, Visualization, Validation, Supervision, Investigation, Conceptualization. **Michael R. Jeng:** Writing – review & editing.

## Ethical clearance

Not required.

## Funding

This research did not receive any specific grant from funding agencies in the public, commercial or not-for-profit sectors.

## Guardian’s consent

We are grateful to the mother and father of the patient spotlighted in this case who have granted permission to share their child’s case and treatment plan.

## Declaration of Competing Interest

None.
